# Venous thromboembolism is rare after total hip and knee joint arthroplasty with long thromboprophylaxis in Finnish fast-track hospitals

**DOI:** 10.1007/s00402-023-04842-w

**Published:** 2023-04-17

**Authors:** Annette M. Moisander, Konsta Pamilo, Antti Eskelinen, Jukka Huopio, Hannu Kautiainen, Anne Kuitunen, Juha Paloneva

**Affiliations:** 1 Department of Anesthesia and Intensive Care, Hospital Nova, Wellbeing Services County of Central Finland, Jyväskylä, Finland; 2 Coxa Hospital for Joint Replacement, Wellbeing Services County of Pirkanmaa, Tampere, Finland; 3 Department of Orthopaedics and Traumatology, Kuopio University Hospital, Wellbeing Servicies County of North Savo, Kuopio, Finland; 4 Primary Health Care Unit, Kuopio University, Folkhälsan Research Center, Helsinki, Finland; 5 Department of Intensive Care, Tampere University Hospital, Wellbeing Services County of Pirkanmaa, Tampere, Finland; 6 Department of Surgery, Hospital Nova, Wellbeing Secvices County of Central Finland, Jyväskylä, Finland; 7 Faculty of Medicine and Health Technologies, University of Tampere, Tampere, Finland

**Keywords:** Hip arthroplasty, Knee arthroplasty, Venous thromboembolism, Fast- track, TKA, THA, TBKA, THR, TKR, Thromboprophylaxis, VTE

## Abstract

**Introduction:**

Pharmacological thromboprophylaxis effectively prevents venous thromboembolism (VTE) after total knee (TKA) and total hip arthroplasty (THA). Less is known about the influence of fast-track arthroplasty on VTE risk. We conducted a register-based study to determine the incidence of VTE after fast-track TKA and THA in Finland using long thromboprophylaxis.

**Materials and methods:**

All primary TKAs and THAs operated during 2015–2016 in 3 fast-track hospitals were identified from the Finnish Arthroplasty Register. Pulmonary embolism (PE) and deep vein thrombosis (DVT) diagnosed in this patient cohort within 90 days of surgery were identified from the Finnish Hospital Discharge Register. The recommended length of thromboprophylaxis was 10 to 14 days for TKA and 28 days for THA during study period.

**Results:**

During the study period, 3 831 THAs, 4 394 TKAs and 286 bilateral TKAs (BTKAs) were performed. Of all these patients, 60% were females. Venous thromboembolism (VTE) incidence within 90 days of surgery was 0.3% (95% CI 0.2–0.4). These VTEs comprised 10 PEs and 15 DVTs. None of the VTE patients´ died within the 90-day period.

**Conclusion:**

VTE incidence is low in Finnish fast-track TKA and THA patients with long thromboprophylaxis.

**Supplementary Information:**

The online version contains supplementary material available at 10.1007/s00402-023-04842-w.

## Introduction

Venous thromboembolism (VTE), including deep vein thrombosis (DVT) and pulmonary embolism (PE), is a feared complication after hip and knee arthroplasty (THA and TKA). Before the routine use of thromboprophylaxis, the incidence of VTE was > 50% [1]. Thromboprophylaxis with low molecular weight heparins (LMWH) and direct oral anticoagulants (DOAC) has reduced this to 1% [2–4].

Fast-tracking was designed to enhance postoperative recovery without compromising quality of care [5–7]. Its goal is to “first do better than faster” eventually leading to shorter length of stay [8–10] When this has been accomplished selected patients can be discharged home on the day of the surgery [11]. Fast-tracking includes preoperative information on discharge criteria to home, multimodal pain management, use of opioid-free spinal anesthesia, local infiltration anesthesia, avoidance of drains and catheters, and mobilization on the day of surgery. Patients are discharged home [7].

A VTE incidence of 0.4% has been reported in Danish fast-track centers [12–14]. These centers use thromboprophylaxis with LMWH or DOAC only during hospitalization if length of stay (LOS) is ≤ 5 days. No compression stockings or intermittent pneumatic compression devices are used [14]. If LOS is > 5 days, thromboprophylaxis is continued after discharge.

In Finland prior to 2022, thromboprophylaxis was recommended to last for 28 to 35 days after THA and 10 to 14 days after TKA. LMWH or DOAC was started 6 to 12 h after the operation. Mobilization on the day of the surgery was encouraged [15]. It was based on the American College of Chest Physicians guidelines for VTE prevention in orthopedic surgery [2]. In it, thromboprophylaxis was recommended to continue for 10 to 14 days with possible extension up to 35 days. LMWH is the drug of choice and should be started either 12 h before or surgery. The use of an IPCD is encouraged during hospitalization.

We conducted a retrospective registry study to define VTE incidence in Finnish fast-track THA and TKA patients with long thromboprophylaxis.

## Materials and methods

This article reports a retrospective register study of consecutive primary total hip, and knee arthroplasty patients operated in 3 Finnish hospitals using a fast-track protocol. Patients were identified from the Finnish Arthroplasty Registry (FAR). All primary arthroplasty patients over age 18 years were identified using the NOMESCO codes (Finnish version) for primary arthroplasty surgery (THA: NFB30, NFB40, NFB50, NFB60, NFB99; TKA: NGB20, NGB30, NGB40 and NGB50 [16] from 1 January 2015 to 31 December 2016. We did not exclude patients with permanent anticoagulation before surgery.

Data from FAR were combined with data from the Finnish Hospital Discharge Register (FHDR). Both registers (FHDR and FAR) are maintained by the National Institute of Health and Welfare. All Finnish hospitals report all surgical procedures to the FHDR and all arthroplasties to the FAR. The FAR includes data on hip and knee arthroplasties since 1980 [17]. The FHDR is one of the oldest discharge registries and dates back to the 1950s [18]. The completeness of the Finnish Arthroplasty Register for primary THA and TKA was over 90% during the years 2015 and 2016 [17]. A systematic review of the quality of the Finnish Hospital Discharge Register (FHDR) evaluated its accuracy and completeness ranging from satisfactory to very good [18].

Patients were grouped by operation type: total hip arthroplasty (THA), total knee arthroplasty (TKA) or bilateral total knee arthroplasty performed under the same anesthesia (BTKA). All the bilateral total hip arthroplasties performed under same anesthesia were excluded owing to their small number (under 50). All the venous thromboses in the three operation groups (THA, TKA and BTKA) were identified from the FHDR using ICD-10 codes, 10^th^ revision [19] (Appendix 2). Causes of death were obtained from the National Causes of death Registry of Statistics Finland.

The catchment area of these 3 academics, publicly funded hospitals (Kuopio University Hospital, Kuopio, Coxa Hospital for Joint Replacement, Tampere, and Hospital Nova, Jyväskylä) contained about one-third of the Finnish population (1 713 738 people) during the study period [20].

All hospitals used a fast-track protocol aimed at early discharge home. This protocol included information on early mobilization, the discharge criteria, and pain management. Pain management was multimodal and local infiltration anesthetic techniques were used with the TKA patients to avoid excessive opioid use. Multimodal pain management included combinations of paracetamol, non-steroidal anti-inflammatory drugs, COX-2 selective pain medication, or gabapentinoids. Surgery was performed under opioid-free low-dose spinal anesthesia. Intraoperative use of drains and catheters was avoided, and intravenous tranexamic acid was administered during surgery. When the discharge criteria of the post-anesthetic care unit (PACU), i.e., stable hemodynamics, pain at a tolerable level, ability to move legs after spinal anesthesia, and no nausea or vomiting, were met, patients are discharged to the ward where they were mobilized the same day, i.e., the day of surgery. Thromboprophylaxis for 28 to 35 days for THAs and 10 to 14 days for TKAs was started 6 to 8 h after surgery. The most used thromboprophylaxis was enoxaparin (40 mg /day) or rivaroxaban (10 mg/day). If pharmacological thromboprophylaxis was contra-indicated, an intermittent pneumatic compression device (IPCD) was used. Patients were discharged to home as soon as the following criteria were met: ability to get out of bed and chair independently; ability to walk 40 m and ascend and descend stairs, if needed; ability to take care of personal hygiene; and the availability of a support person [9].

Data on planned thromboprophylaxis have been recorded in the FAR since 2014. During the years 2015 and 2016, approximately 73% of all the patients with total hip arthroplasties in Finland were prescribed enoxaparin as thromboprophylaxis, 21% were prescribed rivaroxaban, and 4% tinzaparin. The remaining 2% received no planned or recorded thromboprophylaxis or other anticoagulants. Among the patients with total knee arthroplasties, the corresponding proportions were 75, 19, 4, and 2% [21, 22]. Our study also included patients with permanent anticoagulation before arthroplasty. Usually, permanent anticoagulation is discontinued few days before surgery to ensure that spinal anesthesia can be safely administered. Bridging is only used in patients with a hereditary clotting disorder or mechanical heart valve. If possible, platelet inhibitors are substituted with aspirin, which is then continued throughout surgery.

### Statistics

Descriptive statistics are presented as means (SD), medians (IQR) or counts (%). Incidence was estimated by an exact 95% confidence interval (CI) based on a Poisson distribution. Analyses were performed with Stata 15.1 (StataCorp LP; College Station, Texas, USA).

## Results

During study period 8 511 total joint arthroplasties were performed, comprising 3 831 total hip arthroplasties (THAs), 4 394 total knee arthroplasties (TKAs) and 286 bilateral total knee arthroplasties (BTKAs). All BTKAs were performed under the same anesthesia. Of all studied total joint arthroplasties 5 135 (60%) were females. Median length of stay (LOS) was 3 (SD 1) days and median length of uninterrupted institutional care (LUIC) 4 (SD 1) days. A more detailed description of the study population has been reported elsewhere [23].

The incidence of venous thromboembolism (VTE) within 90 days of surgery was 0.3 (95% CI 0.2–0.4). The VTEs consisted of 10 pulmonary embolisms (PEs) and 15 deep vein thromboses (DVTs), and 56% of them occurred in females. PE incidence was 0.1 (CI 0.0–0.2) and DVT incidence 0.2 (CI 0.1–0.3); see Table 1. Thirty-five deaths occurred within 90 days of arthroplasty, none of which were VTE related.

**Table 1:** Distributions and incidences of VTE, PE and DVT by operation group and sex

N (%)	THA	TKA	BTKA	M	F	Total
VTE	13 (52)	12 (48)	0	11 (44)	14 (56)	25 (100)
PE	6 (60)	4 (40)	0	3 (30)	7 (70)	10 (40)
DVT	7 (47)	8 (53)	0	8 (53)	7 (47)	15 (60)
Total	3831 (45)	4394 (52)	286 (3)	3376 (40)	5135 (60)	8511 (100)
Incidence (95% CI)						
VT	0.3 (0.2 to 0.6)	0.3 0.1 to 0.5)	0.0 (0.0 to 1.3)	0.3 (0.2 to 0.6)	0.3 (0.2 to 0.5)	0.3 (0.2 to 0.4)
PE	0.2 (0.0 to 0.3)	0.1 (0.0 to 0.2)	0.0 (0.0 to 1.3)	0.1 (0.0 to 0.3)	0.1 (0.0 to 0.3)	0.1 (0.0 to 0.2)
DVT	0.2 (0.1 to 0.4)	0.2 (0.1 to 0.4)	0.0 (0.0 to 1.3)	0.2 (0.1 to 0.5)	0.1 (0.0 to 0.3)	0.2 (0.1 to 0.3)

*N* number, *THA* total hip arthroplasty, *TKA* total knee arthroplasty, *BTKA* = bilateral knee arthroplasty under same anesthesia, *M* male, *F* female, *CI* confidence interval, *VTE* venous thromboembolism, *PE* pulmonary embolism, *DVT* deep venous thrombosis

No VTEs were diagnosed among the bilateral total knee arthroplasties performed under the same anesthesia. No differences in VTE, PE or DTV incidences between THAs and TKAs or between the sexes were observed.

## Discussion

We found a low (0.3%) incidence of venous thromboembolism (VTE) in Finnish fast-track total hip and knee arthroplasty patients using long thromboprophylaxis. In hospitals not using a fast–track protocol, the incidence of VTE has been reported to range from 1 to 1.2% after regular use of thromboprophylaxis [3]. A systematic review of phase III randomized trials comparing fondaparinux, apixaban, rivaroxaban, or dabigatran with enoxaparin in patients undergoing THA or TKA reported a median VTE-rate of 0.99% [24]. When comparing these results, it is important to bear in mind that patients in phase III clinical trials consists of selected populations. All patients with confounding factors for VTE are excluded, meaning that patients with a history or close family history of venous thrombosis or thromboembolism or with permanent anticoagulation are excluded. The durations of thromboprophylaxis reported in this review, i.e., 10 to 14 days for TKAs and 28 to 40 days for THAs, closely resembled those in our study. Our VTE incidence appears low compared to that in the phase III randomized trials, despite the fact that our patient population was unselected and also included patients at high risk for VTE.

In a large cohort study of 17 582 arthroplasties performed in Danish fast-track arthroplasty hospitals, the incidence of VTE was 0.5% [14]. In this study, thromboprophylaxis was administered during hospitalization only to patients whose hospital stay was 5 days or less. This early discharge population accounted for 95.5% of all the arthroplasties performed between 1 December 2011 and 30 October 2015. Median length of stay (LOS) was 2 days, meaning that the length of thromboprophylaxis was 2 days in this patient group. The incidence of VTE in this early discharge population was 0.4% 90 days after operation. For the remaining patients who were not discharged within 5 days of their operation, median LOS was 7 days and VTE incidence was 2.3%. These patients were older, had more co-morbidities and, according to regional preferences, received thromboprophylaxis for longer than the early discharge population. The length of thromboprophylaxis for the early discharge patients was at least 5 times shorter for TKAs and over 10 times shorter for THAs compared patients with LOS > 5 days. It seems that age, patient disease burden and length of stay have more impact on VTE incidence than length of thromboprophylaxis. This suggests that the individual planning of thromboprophylaxis is important.

A prospective French study from year 2020 of 1949 THA and TKA patients using enhanced recovery program (including multimodal perioperative analgesia, early ambulation within 24 h of surgery, free rehabilitation with limitations on the joint range and weight bearing, discharge preferable home when the patient could walk alone with their pain under control) reported VTE incidence of 0.7% and LOS of 4 days [25]. Thromboprophylaxis with LMWH or DOAC was started on the days of the surgery for most of the patient in this study. However, the length of the thromboprophylaxis was not mentioned. Our results and the French study support the findings of low incidence of VTE reported in early THA and TKA fast -track studies from Denmark [12–14]. All these studies indicate that VTE incidence is low when fast-track or enhanced recovery protocols are used for THA and TKA patients.

As pulmonary embolism (PE) can be fatal, it is a feared complication after THA and TKA. We identified 10 PEs in our study, none of which were fatal. The incidence of PE in our study was 0.1%. Danish fast-track studies have also reported low incidences of PE 90 days after operation, ranging from 0% to 0.21% [12–14]. In all these studies, the incidence of fatal PEs was very low, ranging from 0 to 0.02%, and thromboprophylaxis was administered only during hospitalization. In the study by Husted et al. [12], the mean length of hospital stay was reduced from 7.3 to 3.1 days. This reduction in LOS did not lead to an increase PE incidence.

There is evidence that we cannot totally prevent PEs. Sharrock et al. [26] reported in their review of studies reporting 6 weeks to 3 months all-cause mortality and symptomatic PEs, that use of potent thromboprophylaxis did not diminish occurrence of PE but might increase over-all mortality. Cote et al. [27] reviewed clinical trials assessing prophylactic anticoagulation after TKA. They reported that the rate of PE was relatively constant during 14-year study period although more potent thromboprophylaxis was used at the end of the study period. These studies support the hypothesis that some patients may have a genetic predisposition to develop a PE. And there is need for individual VTE risk assessment.

The incidence of VTE, DVT and PE seems to have decreased over the years, as shown in a large American register study consisting of 363 530 TKA and THA patients [28]. Several reasons for this have been proposed: 1) the development of surgical techniques, which has led to shorter operative times; 2) improved perioperative care and the use of regional anesthesia and more effective postoperative analgesia, which has made earlier postoperative mobilization possible; and 3) shorter duration of hospitalization and more consistent use of thromboprophylaxis [3, 29].

We found no differences in VTE incidence between THAs, TKAs or BTKAs. In a study conducted during the early stages of fast-track arthroplasties in Denmark, Husted et al. [12] reported incidences of DVT and PE of around 0.5% and 0% for all three groups. As in our study, they also found an incidence of DVT or PE of 0% for BTKA patients [12]. It has been reported that DVT and PE incidences are higher after THA than TKA [3]. However, in an American database study of 363 530 patients who received a TKA or THA between 2008 and 2016 the incidence of VTE was 0.6% for THA and 1.4% [28]. The study did not report on the use of thromboprophylaxis or type of care protocol. In this study, the incidence of VTE for THAs did not vary significantly during the study period, although for TKAs it decreased from 3.0% to 1.1%.

Our findings indicate that long thromboprophylaxis protocol efficiently prevents venous thromboembolism. No deaths were caused by pulmonary embolism (PE). One death occurred due to intracranial bleeding, as reported in our previous study of clinically relevant bleeding complications [23]. This would suggest that thromboprophylaxis is safe. However, the relative safety of thromboprophylaxis may be questioned due to the relatively high incidence of clinically relevant bleeding complications found earlier [23]. Most of these bleedings were operational site bleedings that, while not life threatening, can lead to blood transfusions, reoperations, longer length of stay, and postpone mobilization, thereby increasing the possibility of VTE. They also cause discomfort and pain as well as increased use of health care services after discharge and hence increased total costs.

The European Society of Anaesthesiology Intensive Care (ESAIC, previously known as ESA) has issued European guidelines on perioperative venous thromboembolism prophylaxis for day and fast-track surgery [30]. According to these guidelines, thromboprophylaxis during hospitalization can only be used if LOS is 5 days or less in selective fast-track patients at low risk for VTE. Based on these guidelines and the Danish fast-track studies, Finnish recommendation has been updated. Thromboprophylaxis is now planned individually according to the patient’s risk for VTE, and its length depends on specific risk factors (Table 2). Extended 4-week thromboprophylaxis is recommended for patients with 1 major or ≥ 2 minor risk factors. Thromboprophylaxis for 10 to 14 days is recommended for patients regarded as low risk. Thromboprophylaxis for low risk, fast-track patients can be used only during hospitalization if length of stay is under 5 days [31]. The very low incidence of VTE in this study supports this transition towards shorter and individualized thromboprophylaxis.

**Table 2:** Risk factors

Major risk	Minor risk
Active cancer or in treatment cancer	Age > 60 years
Previous venous thromboembolism	Body mass index > 40
Venous thromboembolism in 1^st^ degree family members	Immobilization before surgery (non- walking, 4 days)
Operation time > 120 min	Chronic venous insufficiency
Hereditary clotting disorder	

A limitation of this study is the quality of the registers used. However, the completeness of the registries used are reportedly good [18, 32, 33]. There is a possibility that, owing to the use of multiple codes with vague definitions, the ICD-10 code recorded in the FHDR might not be accurate enough to detect all cases of deep vein thrombosis. However, we consider it reliable with respect to pulmonary embolism as only 2 codes are possible.

A strength of our study is that we studied an unselected sample of fast-track arthroplasty patients with or without permanent anticoagulation before surgery. Patients who did not receive any anticoagulation due to an underlying illness might also have been included. Thus, our study is representative of a fast-track arthroplasty population. Moreover, all three study hospitals used long thromboprophylaxis during the study period. From 2020 onwards, two of three hospitals have been using shorter, more individualized thromboprophylaxis. This shorter thromboprophylaxis protocol resembles the new recommendation issued by the Finnish Arthroplasty Society in 2022 [31]. Another strength of our study is that because the recording of surgical procedures in the FDHR is mandatory, it includes those performed in both public and private healthcare facilities, thereby providing good coverage of VTEs diagnosed after THA and TKA. Causes of deaths are also recorded with high reliability.

## Conclusion

**T**he incidence of VTE is very low in fast-track THA and TKA patients when long thromboprophylaxis is used. Combining the present findings with those of our previous study on clinically relevant bleeding complications [23], shorter thromboprophylaxis could also be a possibility for fast-track TJA patients.

### Electronic supplementary material

Below is the link to the electronic supplementary material.

Supplementary file1 (DOCX 16 KB)

Supplementary file2 (DOCX 14 KB)

## Data Availability

For data availability contact the correponding author.

## Appendix 1 NOMESCO codes – Finnish version

NFB30 Primary total prosthetic replacement of hip joint not using cement.

NFB40 Primary total prosthetic replacement of hip joint using hybrid technique.

NFB50 Primary total prosthetic replacement of hip joint using cement.

NFB60 Demanding prosthetic replacement of hip.

NFB99 Other primary prosthetic replacement of hip joint.

NGB20 Primary total prosthetic replacement of knee joint without patellar part—sliding prosthesis.

NGB30 Primary total prosthetic replacement of knee joint without patellar part—connected prosthesis.

NGB40 Primary total prosthetic replacement of knee joint with patellar part sliding prosthesis.

NGB50 Primary total prosthetic replacement of knee joint with patellar part connected prosthesis.

## Appendix 2 ICD–10 codes used

**Table:**

	ICD–10 code
Pulmonary embolism	I26.0, I26.9
Venous thromboembolism	I80.1–9, I81, I82
