# Supplementary material for: Venous thromboembolism is rare after total hip and knee joint arthroplasty with long thromboprophylaxis in Finnish fast-track hospitals
Source: Arch Orthop Trauma Surg. 2023 Apr 17;143(9):5623–9. doi: 10.1007/s00402-023-04842-w (PMC10449718; doi:10.1007/s00402-023-04842-w)
Supplement: Supplementary file 1 — Supplementary file1 (DOCX 16 KB) [file 402_2023_4842_MOESM1_ESM.docx]

**Appendix I NOMESCO codes – Finnish version**

NFB30 Primary total prosthetic replacement of hip joint not using cement

NFB40 Primary total prosthetic replacement of hip joint using hybrid technique

NFB50 Primary total prosthetic replacement of hip joint using cement

NFB60 Demanding prosthetic replacement of hip

NFB99 Other primary prosthetic replacement of hip joint

NGB20 Primary total prosthetic replacement of knee joint without patellar part – sliding prosthesis

NGB30 Primary total prosthetic replacement of knee joint without patellar part – connected prosthesis

NGB40 Primary total prosthetic replacement of knee joint with patellar part ­ sliding prosthesis

NGB50 Primary total prosthetic replacement of knee joint with patellar part ­ connected prosthesis
