# Supplementary material for: Venous thromboembolism is rare after total hip and knee joint arthroplasty with long thromboprophylaxis in Finnish fast-track hospitals
Source: Arch Orthop Trauma Surg. 2023 Apr 17;143(9):5623–9. doi: 10.1007/s00402-023-04842-w (PMC10449718; doi:10.1007/s00402-023-04842-w)
Supplement: Supplementary file 2 — Supplementary file2 (DOCX 14 KB) [file 402_2023_4842_MOESM2_ESM.docx]

**Appendix II ICD–10 codes used**

|  | ICD–10 code |
| --- | --- |
| Pulmonary embolism | I26.0, I26.9 |
| Venous thromboembolism | I80.1-9, I81, I82 |
